# Social support, perceived stigma, and depression among PLHIV on second-line antiretroviral therapy using structural equation modeling in a multicenter study in Northeast Ethiopia

**DOI:** 10.1186/s13033-022-00536-9

**Published:** 2022-06-13

**Authors:** Shambel Wedajo, Getu Degu, Amare Deribew, Fentie Ambaw

**Affiliations:** 1 School of Public Health, CMHS, Wollo University, Dessie, Ethiopia; 2 School of Public Health, CMHS, Bahir Dar University, Bahir Dar, Ethiopia; 3 Country Director, Nutrition International (NI) in Ethiopia, Addis Ababa, Ethiopia

**Keywords:** Social support, Depression, Stigma, SEM, HIV/AIDS, Second-line ART

## Abstract

**Background:**

Depression has a multitude of clinical and public health consequences for HIV patients. The magnitude of HIV patients who failed first-line antiretroviral treatment and switched to second-line therapy is becoming a growing public health concern. However, unlike first-line therapy, to date, little attention has been given to mental health problems in such patients, particularly in the era of the COVID-19 pandemic. Thus, this research was conducted to determine the magnitude of depression and its determinants among HIV patients on second-line antiretroviral therapy.

**Methods:**

A multi-centered cross-sectional study was conducted on 714 HIV patients on second-line therapy who were selected via systematic random sampling. Data were collected in personal interviews as well as document reviews. The nine-item patient health questionnaire score was used to assess depression, while the three-item Oslo Scale was used to assess social support. The associations between exogenous, mediating, and endogenous variables were identified simultaneously using structural equation modeling. Statistical significance was declared at a P-value less than 0.05, and the effect sizes were presented using 95% CI.

**Results:**

Depression was reported in 27.7% of HIV patients on second-line therapy [95% CI: 24.7–31.1%]. Social support has a direct [$\widehat{\beta }$ = − 0.9, (95% CI: − 1.11 to − 0.69)] and indirect [$\widehat{\beta }$ = − 0.22, (95% CI: − 0.31 to − 0.13)] negative effect on depression. Perceived stigma was a mediator variable and significantly associated with depression [$\widehat{\beta }$ = 0.40, (95% CI: 0.23–0.57)]. Co-morbid illness [$\widehat{\beta }$ = 0.49, (95% CI: 0.35–0.63)], high viremia [$\widehat{\beta }$ = 0.17, (95% CI: 0.08–0.26], moderate and high-risk substance use [$\widehat{\beta }$ = 0.29, (95% CI: 0.18–0.39)], and not-workable functional status [$\widehat{\beta }$ = 0.2, (95% CI: 0.1–0.31)] were all positively associated with depression.

**Conclusions:**

This study revealed that there was a high prevalence of depression among HIV patients on second-line antiretroviral therapy. Social and clinical factors were associated with depression risk. As a result, screening, prevention, and control strategies, including psychosocial support, should be strengthened in routine clinical care.

## Introduction

Globally, 27.5 million people living with HIV (PLHIV) have accessed antiretroviral therapy (ART) by the end of 2020 [1]. However, currently, the magnitude of HIV patients on second-line antiretroviral therapy is becoming a growing public health concern. A considerable number of patients worldwide had experienced first-line treatment failure and switched to second-line antiretroviral treatment [2]. In Ethiopia, the proportion of people living with HIV on second-line antiretroviral therapy is also a rising issue; according to a systematic review, 15.9% (11.6–20.1%) of PLHIV had failed their first-line regimen [3]. Treatment failure is characterized by a worsening of HIV clinical symptoms while on antiretroviral treatment, and those patients will be switched to second-line antiretroviral therapy [4].

PLHIVs are disproportionately affected by mental health problems [5].“Depression is a common mental health problem characterized by sadness, loss of interest or pleasure, feelings of guilt or low self-worth, disturbed sleep or appetite, feelings of tiredness, and poor concentration” [6]. According to the Ethiopian national health survey, the prevalence of depression in the general population was 9.1% (95CI%: 8.39–9.9%) [7]. In contrast, a systematic review conducted in Ethiopia revealed that the pooled prevalence rate of depression in PLHIV on first-line antiretroviral was 36.65% (25.48, 47.82%) [8]. Furthermore, in the era of the COVID-19 pandemic, PLHIVs not only have a higher risk of severe illness [9], but they also may have an increased risk of depression, which is associated with stress, social isolation, and stigma [10].

Depression and HIV infection have a bidirectional relationship [4]. Depression can result in worsening HIV-related treatment outcomes and compromise the overall quality of life, including poor medication adherence, immunological failure, and the development of severe opportunistic infection. Consequently, increase the chance of subsequent treatment failure and the development of drug-resistant strains. Depression can also raise the likelihood of substance abuse and other high-risk behaviors including the risk of HIV acquisition [11]. Similarly, HIV infection causes depression as a result of coping with the diagnosis, worsening clinical symptoms, stigma, social rejection, co-existing poverty, and the adverse effects of certain antiretroviral medications [4].

Even though HIV and depression are linked, very little is known about the magnitude of depression among HIV patients who were receiving second-line antiretroviral therapy, especially during the COVID-19 pandemic. Besides, some symptoms of depression and HIV are mimicked; consequently, depression in PLHIV might be under-recognized and under-treated.

The risk factors for depressive symptoms are interconnected, and most previous studies have used the univariate analysis method to address these issues. However, this method of analysis has limitations in terms of identifying the direct and indirect effects of covariates, as well as failing to account for measurement errors for latent constructs such as depression, social support, and internal and perceived stigma. Furthermore, factors which lead to depressive symptoms are heterogeneous, and local evidence is required for context-based decision making and intervention during the COVID-19 pandemic. Social distancing and other mitigation strategies in the prevention of COVID-19 have a considerable effect on social support and mental health problems [12]. To mitigate COVID-19, HIV, and mental health issues, it is necessary to generate cultural and context-based evidence.

Hence, based on the above-identified gaps, we conducted a multi-centered cross-sectional study to determine the magnitude of depression and to investigate the association between depression symptoms and socio-demographic, clinical, social support, and internal and perceived stigma among PLHIV on second-second antiretroviral therapy. To investigate such factors, the Structural Equation Model (SEM) was employed.

## Methods

### Study setting

This study was conducted in twenty public health facilities that currently provide second-line therapy in the eastern Amhara region, northeast Ethiopia. Of those facilities, six were hospitals, and fourteen were health centers. According to Ethiopia's national HIV/AIDS guideline, health centers having greater than 200 HIV patient loads are allowed to initiate second-line treatment. Eastern Amhara is a high-burden area in the region [13, 14]. Currently, 2332 PLHIV are attending the second-line program in the above health facilities.

In Ethiopia, the current standard second-line antiretroviral therapy consists of a combination of three ARV drugs (at least two of which are new to the patient); two Nucleoside Reverse Transcriptase Inhibitors (NRTIs) as a backbone; Lamivudine (3TC) and Abacavir (ABC), or Zidovudine (ZDV) or Tenofovir (TDF) and one protease Inhibitors (PIs); Lopinavir/ ritonavir (LPV/r) or Atazanavir /ritonavir (ATV/r) [4, 15]. Those PIs drugs are safer, more effective in viral suppression, and have a lower risk of resistance [16, 17].

Moreover, all facilities use the same documentation and reporting system, and HIV data are handled by SMART care, ART registration/logbook/, and chronic ART follow-up form [15]. At every clinical visit, these registers are updated. PLHIV is currently scheduled every 3 months. Patients will be assessed for nutritional status, opportunistic infections, medication adherence, drug side effects, and refilled with ART and other preventative medications at each appointment. Antiretroviral treatment success is monitored using clinical, immunological, and viral load (VL) assessment, in which viral a load test is done after initiation of ART at 6 months, 12 months, and every 12 months and serves as a gold standard monitoring tool [4, 15].

### Study design and period

A multi-centered cross-sectional study was conducted in the Eastern Amhara region, Northeast Ethiopia, from December 2020–to February 2021.

### Source and study population

HIV patients who were receiving second-line antiretroviral therapy and following their HIV care and treatment in the eastern Amhara region were considered a source population, whereas those who were available during the data collection period were considered a study population.

### Variables and measurements

The primary outcome variable in this study was depression, which was measured using the nine-item Patient Health Questionnaire (PHQ-9) [18]. Each question requires participants to rate the frequency of a depressive symptom experienced in the two weeks before evaluation. The total score ranges from 0 to 27. The severity of depression symptoms was divided into five categories: not depressed (5 points), mild depression (5–9 points), moderate depression (10–14 points), moderately severe depression (15–19 points), and severe depression (20–27 points). To determine the prevalence of clinical depression, patients having depression scores of ten or above were considered as having clinical depression. This PHQ-9 items depression screening tool was validated in the Ethiopian context [19].

Perceived and internalized stigma were measured by six and eight items on the HIV Stigma Scale [20, 21]. Items were evaluated using a four-point Likert scale (strongly disagree, disagree, agree, and strongly agree), with higher scores indicating higher levels of stigma. The total score of the scale for PS and IS ranged from 6–24 and 8–32, respectively.

In this study, social support is expected to have a direct and indirect effect on depression, which was measured using the three**-**item Oslo Scale [22]. The Oslo Scale's total score ranges from 3 to 14, with the higher value indicating strong social support. For descriptive purposes, the social support scale was divided into three broad categories: "poor support" (3–8), "moderate support" (9–11), and "strong support" (12–14). For SEM analysis, the row social support score was used as a continuous scale.

The risk of substance use was measured using the WHO Alcohol, Smoking, and Substance Involvement Screening Test (ASSIST) (V.3.1), which consists of seven items for each of alcohol, khat use, and tobacco products [23]. Participants were then classified as having low, moderate, or high-risk substance use depending on their score.

**Fig. 1:**
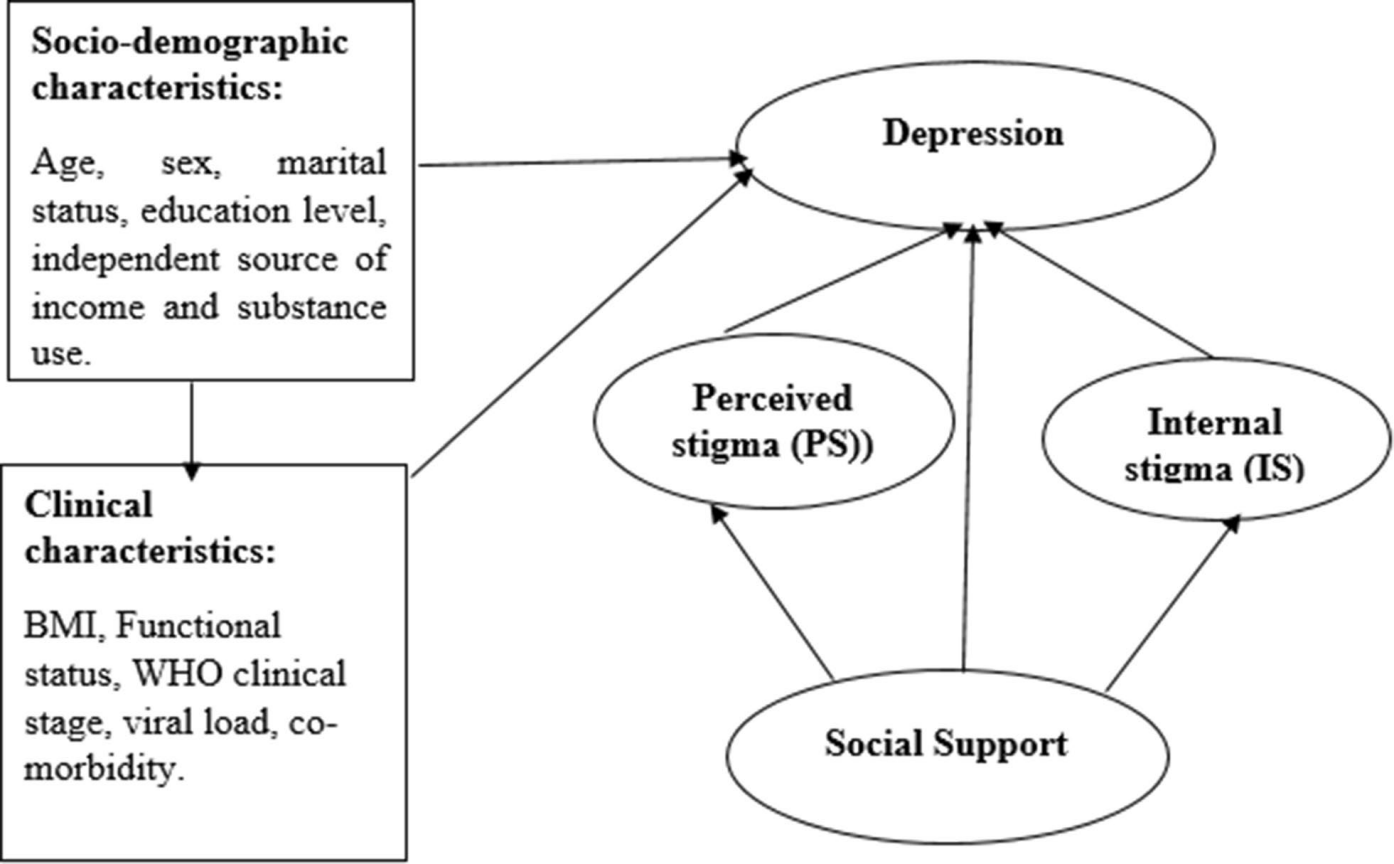
A hypothesized model for factors associated with depression among people living with HIV on second-line antiretroviral therapy (oval and rectangular shape variables represent latent and observed variables, respectively)

The socio-demographic characteristics include age, sex, marital status, education level, independent source of income, and substance use. Similarly, the following patient clinical characteristics were included: Body mass index (BMI), functional status, WHO-clinical stage, viral load, medication adherence, and comorbidity (Fig. 1). An individual's comorbidity status was assessed using a self-report method. Co-morbid NCDs are defined as a patient who has one or more of the following confirmed non-communicable illnesses (NCDs): diabetes, cardiovascular disease, hypertension, and cancer. Medication adherence was assessed using a self-report method taken from the patient's medical records. Patients who were taking 95% of the prescribed antiretroviral medication were considered to have optimal adherence unless classified as not having optimal medication adherence [15]. Patients with viral load measurements below 1000 copies/mL are considered as low viremia unless they have been classified as having high viral load (high viremia) [4]. Two criteria were used to classify CD4 cells. Healthy individual CD4 cell counts range from 450 to 1500. The risk of opportunistic infection begins in HIV patients when CD4 cell counts are less than 450. Hence, based on the above two concepts, 450 cells/mm3 was used to categorize the last CD4 cell measurement [4, 15].

### Sample size

The basic rule of thumb for determining the minimum required sample size for structural equation modeling is 5 to 20 times the number of free parameters to be estimated in the hypothesized model [24]. A 1:10 ratio of free parameters to be estimated to respondents was used to calculate the required sample size to meet the study's objectives. This implies that there should be ten responders for each free parameter in the hypothesized model. As a result, the required sample size was determined to be 670, based on the 67 parameters to be estimated based on the postulated model (Fig. 1). The final sample size, after adding 10% for non-response, was computed as 737.

### Sampling procedure

A systematic random sampling method was used to select the sample individuals. First, the sampling frame was secured in each facility by reviewing the updated ART registration logbook. Then, based on the number of patient loads, samples were proportionally allocated to each health facility. Finally, using the systematic random sampling method, sample clients were recruited every third interval while the clients came for routine follow-up.

### Data collection

Data were collected using a structured questionnaire via face-to-face interviews by trained degree nurses at the ART outpatient department (ART-OPD). A questionnaire was prepared in the local language and data were collected for a three-month duration while the patient came for a routine clinical visit. Moreover, document reviews are also done to extract patient clinical profiles. The extraction sheet was prepared per the national consolidated antiretroviral guidelines [15].

### Data quality management

To ensure the quality of the data, the questionnaire was prepared by considering existing literature and guidelines. Furthermore, validated tools were used to measure latent constructs. The questionnaire was also pretested, and necessary modifications were made. Data collectors were trained on the objective of the study, the content of the questionnaire, and, in general, how to collect the required data. The data collection procedures were closely supervised, and on-site feedback was given.

### Data analysis

Data were cleaned and entered into the EpiData Version 3.1 software. Then, exported to Stata version 14 for further analysis. For categorical variables, frequency (%) was computed. For a continuous variable, first, distributional assumptions were checked using the Kolmogorov–Smirnov and Shapiro–Wilk test. Then, for normally and skewed distributed continuous variables, mean with standard deviation (SD) and median (interquartile range, IQR) were employed, respectively.

The hypothesized association between various exogenous and endogenous or mediating variables was verified using the Structural Equation Model (SEM). The effect size was presented using standardized and unstandardized beta coefficients, and statistical significance was declared at a P-value less than 0.05. The analysis began with the hypothesized model (Fig. 1), and modifications were made iteratively by comparing model fit indices and information criteria of each model fitted, then adding path links or including mediator variables if the path coefficient is statistically significant and theoretically supported. Finally, an over-identified model with the best model fit indices and the smallest amount of information was kept (Fig. 1). The direct, indirect, and total effects were calculated using a nonlinear combination of estimator techniques when the mediation effect was present.

## Results

### Socio-demographic characteristics

Out of 737 approached sample patients, 714 (96.87%) agreed and gave their consent to participate in this study. Of those surveyed, 468 (65.5%) lived in urban areas, 400 (56%) were female, and 342 (47.9%) were married. Concerning the source of income, 511 (71.6%) participants had an independent source of income. Similarly, of the 714 participants, 281(39.4%) had not attained formal education, and 355 (49.7%) were orthodox religious followers. The median (IQR) age and year on antiretroviral therapy of participants were 37 (30–45) years and 10 (8–13) years, respectively (Table 1).

**Table 1:** Socio-demographic characteristics of PLHIV on second-line antiretroviral therapy in the Eastern Amhara region, Northeast Ethiopia, December 2020–February 2021 (n = 714)

Socio-demographic characteristics	Total (714)	Depression (198)
	N (%)	N (%)
Sex		
Female	400 (56)	122 (30.5)
Male	314 (44)	76 (24.2)
Residence		
Urban	468 (65.5)	122 (26.1)
Rural	246 (34.5)	76 (30.9)
Marital status		
Married	342 (47.9)	78 (22.8)
Single	149 (20.9)	52 (34.9)
Divorce	142 (19.9)	53 (37.3)
Widowed	81 (11.3)	15 (18.5)
Religion		
Orthodox	355 (49.7)	102 (28.7)
Muslim	346 (48.5)	93 (26.9)
Protestant	10 (1.4)	2 (20)
Catholic	3 (0.4)	1 (33.3)
Educational level		
Not formally educated	281 (39.4)	79 (28.1)
Primary school (Grade 1–8)	268 (37.5)	69 (25.7)
Secondary school (9–12)	109 (15.3)	28 (25.7)
Above 12 grade (12.^+^ grade)	56 (7.8)	22 (39.3)
Independent source of income		
Yes	511 (71.6)	122 (23.9)
No	203 (28.4)	76 (37.4)
Risk of substance use		
Low-risk substance use	574 (80.4)	109 (19.0)
Moderate and high-risk substance use	140 (19.6)	89 (63.6)
Age, median (IQR) (year)	37 (30–45)	32 (27–44.5)
Year on antiretroviral therapy, median (IQR) (year)	10 (8–13)	10 (7–12)

### Clinical characteristics

Out of 714 study participants, 545 (76.3%) had a BMI of >  = 18.5 kg/m^2^, while 540 (75.6%) participants had viral load measurements below 1000 copies/ml. Similarly, 685 (95.9%) and 608 (85.2%) participants were not in advance of the WHO clinical stage and workable functional status, respectively. Regarding medication adherence and comorbidity status, 679 (95.1%) patients had optimal adherence and 63 (8.8%) patients had a comorbid disease. TDF-3TC-ATV/r (361 (50.6%)) and AZT-3TC-ATV/r (338 (47.3%) were the most prescribed second-line antiretroviral regimens (Table 2).

**Table 2:** Clinical characteristics of PLHIV on second-line antiretroviral therapy in the Eastern Amhara region, Northeast Ethiopia, December 2020–February 2021 (n = 714)

Clinical characteristics	Total (714) N (%)	Depression (198) N (%)
BMI		
> = 18.5 kg/m^2^	545 (76.3)	146 (26.8)
< 18.5 kg/m^2^	169 (23.7)	52 (30.8)
Functional status		
Workable	608 (85.2)	134 (22.0)
Not workable	106 (14.8)	64 (60.4)
WHO clinical stage		
I and II	685 (95.9)	179 (26.1)
III and IV	29 (4.1)	19 (65.5)
Last HIV viral load measurement		
High viremia	540 (75.6)	110 (20.4)
Low viremia	174 (24.4)	88 (50.6)
Last CD4 cells measurement		
< = 450 cell/mm^3^	603 (84.5)	174 (28.9)
> 450 cell/mm^3^	111 (15.5)	24 (21.6)
Medication adherence		
Optimal adherence	679 (95.1)	174 (25.6)
Not optimal adherence	35 (4.9)	24 (68.6)
Comorbidity status		
No	651 (91.2)	156 (24.0)
Yes	63 (8.8)	42 (66.7)
Second-line ART		
TDF-3TC-ATV/r	361 (50.6)	91 (25.2)
AZT-3TC-ATV/r	338 (47.3)	100 (29.6)
TDF-3TC-LPV/r	11 (1.5)	6 (54.5)
AZT-3TC-LPV/r	4 (0.6)	1 (25)

### Depression, social support, and stigma measurement

The prevalence of clinical depression among HIV patients on second-line therapy was 27.7% (95% CI 24.7–31.1%). Out of 714 participants, 47.1% (336) had no signs of depression, while 25.2%, 19.9%, 5.7%, and 2.1% of patients had mild, moderate, moderately severe, and severe signs of depression, respectively. Concerning social support, 24.8% (177), 51.3% (366), and 23.9% (171) of participants had poor, moderate, and strong social support, respectively. The mean (± SD) perceived stigma score of participants was 11.2 ± 4.1. The mean (± SD) internal stigma score of participants was 14.2 ± 4.2.

### Correlations between depression, social support, perceived stigma, and other covariates

The relation between depression with social support and perceived stigma was statistically significant with Pearson correlation coefficient (r) measurement (r = − 0.63, P-value < 0.001) and (r = 0.57, P-value < 0.001), respectively. Social support was negatively correlated with perceived stigma (r =− 0.48, P-value < 0.001) (Table 3).

**Table 3:** Correlation between depression, social support, perceived stigma, and other covariates

	Depression (Dep)	Social support (SS)	Perceived stigma (PS)	Functional status (Fun)	High viremia (VL-sup)	Comorbidity (CoM)	Substance use (Sub-use)
Depression	1						
SS	− 0.6268	1					
PS	0.5737	− 0.4808	1				
Fun	0.3271	− 0.2146	0.281	1			
VL-sub	0.3132	− 0.2793	0.2395	0.1942	1		
CoM	0.2973	− 0.1411	0.1629	0.0784	0.0534	1	
Sub-use	0.3998	− 0.3065	0.3178	0.2700	0.1716	0.0329	1

*SS* social support, *PS* perceived stigma, *Fun* functional status, *VL-sub* last viral load measurement, *CoM* comorbidity with chronic diseases, *SubUse* risk of substance use

### Measurement model

**Fig. 2:**
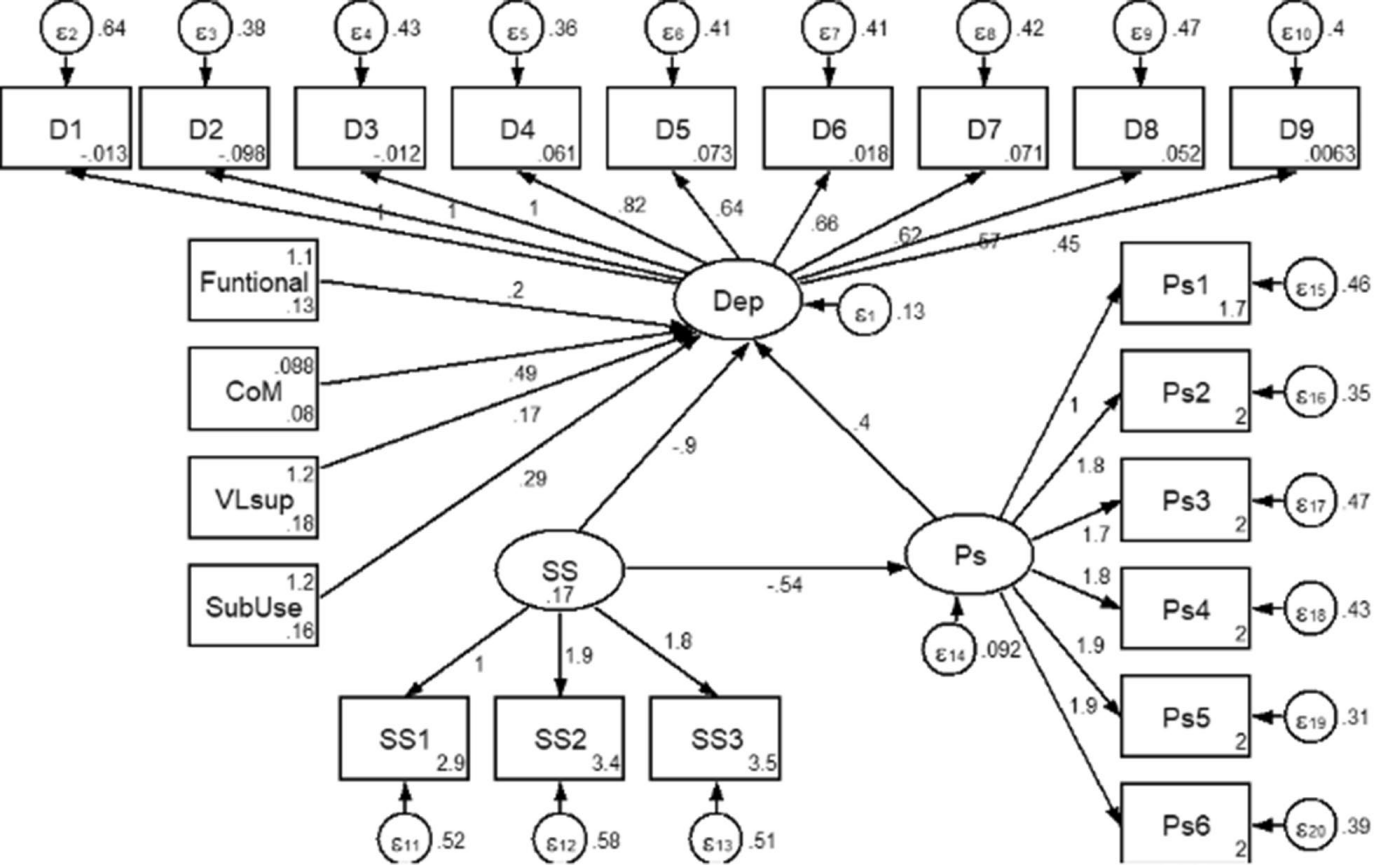
Structural Equation Modeling on examining the association of social support, perceived stigma, and other predictors with depression symptoms. *Dep* depression, *SS* social support, *Ps* perceived stigma, *VLsub* last viral load measurement, *CoM* comorbidity with chronic diseases, *SubUse* risk of substance use

Confirmatory Factor Analysis (CFA) revealed that all the factor loadings for latent variables were statistically significant at a P-value less than 0.001. Furthermore, an evaluation of model fitness was made. It revealed that the final model was a good fit at Root Mean Square Error of Approximation (RMSEA) = 0.07, Standardized root mean square residual (SRMR) = 0.04, and a Comparative Fit Index (CFA) = 0.93 value (Fig. 2).

### Factors associated with depression (direct and indirect effects)

Social support has a direct and indirect negative effect on depression. The direct effect of social support was that as a patient's social support score increased by one unit, their depression score fell by 0.90 factor on average while holding the effect of other covariates in the model constant [$\widehat{\beta }$ = − 0.9, (95% CI: − 1.11 to − 0.69)]. Furthermore, perceived stigma moderated the effect of social support on depression [$\widehat{\beta }$ = − 0.22, (95% CI: − 0.31 to − 0.13)]. Overall, with one unit increase in social support score, on average depression symptom score fell by 1.12 factors, while all other variables in the model remained constant [$\widehat{\beta }$ = − 1.12 (95% CI: − 1.34 to − 0.90)].

Similarly, as the patient-perceived stigma score increased by one unit, the average depression symptom score increased by 0.40, while holding the effect of other covariates in the model constant [$\widehat{\beta }$ = 0.40, (95% CI: 0.23–0.57)].

Patients with a non-workable functional status had a 0.2 higher average depressive symptom score than those with a workable functional status [$\widehat{\beta }$ = 0.2, (95% CI: 0.1–0.31)]. Patients with a high HIV viral load and those who had comorbid disease had an average depression score of 0.17 and 0.49 points higher than their counterparts, respectively. Patients at moderate and severe risk of substance use have a 0.29 higher risk of depression than low-risk patients [$\widehat{\beta }$ = 0.29, (95% CI: 0.18–0.39)] (Table 4).

**Table 4:** Direct and indirect effect of socio-demographic, clinical, and perceived stigma on depression symptoms among PLHIV on Second-line antiretroviral therapy in Eastern Amhara region, Northeast Ethiopia, December 2020–February 2021 (n = 714)

	Estimates ($\widehat{\beta }$)	Standardized estimate	SE of $\widehat{\beta }$	95% CI
*Covariates*				
Social support → depression	− 0.90	− 0.58	0.11	− 1.11 to − 0.69
Social support → perceived stigma	− 0.54	− 0.6	0.07	− 0.66 to − 0.41
Perceived stigma → depression	0.40	0.24	0.09	0.23–0.57
An indirect effect of social support				
SS → PS → depression	− 0.22	− 0.14	0.05	− 0.31 to − 0.13
The total effect of social support				
SS → depression	− 1.12	− 0.72	0.11	− 1.34 to − 0.90
*Other covariate effects on depression*				
Functional status: not workable	0.2	0.11	0.06	0.1–0.31
Comorbidity status: being comorbid	0.49	0.22	0.07	0.35–0.63
Last HIV viral load measurement				
High viremia (VL > = 1000copies/ml)	0.17	0.11	0.05	0.08–0.26
Risk of substance use				
Moderate and severe risk of substance use	0.29	0.18	0.05	0.18–0.39

## Discussion

The purpose of this study was to determine the magnitude of depression and its determinants among PLHIV on second-line antiretroviral therapy. And found that more than one in four HIV patients had experienced clinically significant depression. Poor social support, high perceived stigma, comorbidities, substance use, uncontrolled viremia, and non-workable functional status were factors significantly associated with depression.

The prevalence rate of clinically significant depression in this study was 27.7%. This finding is higher than Ethiopia's national health survey depression prevalence rate (9.1%), which was conducted on the general population [7] as well as other studies done on HIV patients [25–27]. In contrast, the finding of this study is lower than some other studies [28–32]. This variation could be due to the difference in depression screening tools used in different research. Besides, socio-demographic and cultural variation might have a role in the variability of depression prevalence from place to place. In general, this finding confirms that a considerable number of HIV patients are affected by depression. This may have multi-face implications at the individual, public, and policy levels. Hence, screening, prevention, and control strategies including psychosocial support should be strengthened in routine HIV clinical care by integrating with WHO’s mental health Gap Action Programme (mhGAP) [33].

The likelihood of depression was found to be negatively related to social support. This association is supported by many studies [8, 31, 34]. Perceived stigma, on the other hand, was positively correlated with depression, which is consistent with prior research findings [8, 21, 31]. Patients who receive social support from family, friends, and other concerned individuals can develop a stronger sense of self-worth [35], gain strength and courage, and feel safe and supported during their illness [36]. Social support also improves medication adherence [37–39], clinical outcomes, and reduces HIV/AIDS stigma [38], all of which may reduce the risk of depression. Furthermore, social support reduces stress, fear, and the risk of depression associated with COVID-19 [40–42]. Generally, this study implies that social support plays a pivotal role in averting mental health problems. Hence, support from family and friends should be encouraged at the societal level while keeping COVID mitigation strategies.

Being non-workable functional status and being comorbid with non-communicable diseases (NCDs) were positively associated with the risk of depression. This finding is consistent with the findings of other studies [25, 32]. Prior literature showed that a significant number of PLHIV had developed non-communicable diseases such as cardiovascular disease, diabetes mellitus, and cancer [43, 44]. Co-occurrence of NCDs in people living with HIV increases pill burden, worsens chronic pain, impaired diet, and disrupts sleep patterns. This also further increases the risk of depression and other mental health problems. Moreover, being unproductive and dependent on others in daily living aggravates the occurrence of depression. Hence, NCD intervention packages should be integrated and delivered with chronic HIV care at the ART clinic.

Uncontrolled viremia and the risk of depression were also positively correlated, which is supported by other studies [45, 46]. When viremia is uncontrolled while on second-line antiretroviral therapy, patients may be concerned about developing drug resistance, treatment failure, and clinical worsening. As a result, depression and other mental health concerns will become more common. Thus, patient-centered psychological counseling, routine viral load monitoring, and enhanced adherence support should be strengthened for high viral load patients.

Substance use was linked to an increased risk of depression, which is supported in prior studies [7, 26, 47]. Patients on second-line antiretroviral therapy may experience psychological stress, particularly during the COVID-19 pandemic, and substance use might provide temporary relief from mental distress. Substance abuse, on the other hand, leads to depression and other mental health issues over time [48, 49]. Depression, in turn, may increase the chance of substance use. This finding suggests that screening for risk of substance use should be promoted in routine HIV clinical care to reduce the burden of depression in HIV patients.

### Strength of the study

First, the study is the first of its kind in Ethiopia to provide first-hand information to improve depression in HIV patients on second-line therapy. Second, this study examines the direct, and indirect effects of covariates using SEM analysis while controlling measurement errors for latent variables. Third, it was conducted with large sample size and considered social and clinical variables simultaneously. Lastly, the findings of this study can be generalized to other low-income areas where similar WHO HIV treatment modalities are used.

### Limitation of the study

This study failed to examine depression episodes across time. As a result, longitudinal research should be considered as a possible solution that allows seeing depression episodes across time after the commencement of ARV. The other limitation was related to the depression screening tool, which is not a diagnostic tool. Self-reported adherence measurement may introduce an overestimation of adherence secondary to social desirability and intentional recall bias. Similarly, risk of substance use and stigma assessment tools may also have social-desirability and intentional recall bias. Comorbidity is measured via self-reported whether a patient has confirmed diabetes mellitus, cardiovascular disease, hypertension, and cancer. This may undermine the proportion of comorbid cases.

## Conclusions

This study revealed that there was a high prevalence of depression among HIV patients taking second-line antiretroviral therapy. Social support has a direct and indirect effect on depression. Perceived stigma mediates the effect of social support on depression. Having comorbid NCDs, uncontrolled viral load, substance use, and not-workable functional status were all found to be significant predictors of depression in PLHIV.

## Data Availability

The materials and datasets used and analyzed in the current study are available from the corresponding author upon reasonable request.

## Abbreviations

ART: Antiretroviral therapy
PLHIV: People living with HIV
VL: Viral load
BMI: Body Mass Index
IQR: Interquartile range
SEM: Structural Equation Model
PHQ-9: Nine items patient health questionnaire
COVID-19: Coronavirus Disease 2019
NCD: Non-communicable disease (NCDs)

## References

[1] UNAIDS. FACT SHEET 2021 global HIV statistics. End. AIDS epidemic, no. June, pp. 1–3, 2021.

[2] Estill J (2016). The need for second-line antiretroviral therapy in adults in sub-Saharan Africa up to 2030: a mathematical modeling study. Lancet HIV.

[3] Endalamaw A, Mekonnen M, Geremew D, Yehualashet FA, Tesera H, Habtewold TD (2020). HIV/AIDS treatment failure and associated factors in Ethiopia: meta-analysis. BMC Public Health.

[4] World Health Organization. Consolidated guidelines on the use of antiretroviral drugs for treating and preventing HIV infection. 2016. 27466667

[5] Secretariat. HIV/AIDS and mental health: report by the,” 2008.

[6] World Federation for Mental health. Depression : a global crisis. Companies, Int Trade Hum Rights, pp. 1–40, 2010.

[7] Hailemariam S, Tessema F, Asefa M, Tadesse H, Tenkolu G (2012). The prevalence of depression and associated factors in Ethiopia: findings from the National Health Survey. Int J Ment Health Syst.

[8] Amare T, Getinet W, Shumet S, Asrat B (2018). Prevalence and associated factors of depression among PLHIV in Ethiopia : systematic review and meta-analysis, 2017. Hindawi AIDS Res Treat.

[9] Chenneville T, Gabbidon K, Hanson P, Holyfield C (2020). The impact of COVID-19 on HIV treatment and research: a call to action. Int J Environ Res Public Health.

[10] Mi T (2021). Mental health problems of HIV healthcare providers during the COVID-19 Pandemic: the interactive effects of stressors and coping. AIDS Behav.

[11] Bertagnolio S et al. Clinical features and prognostic factors of COVID-19 in people living with HIV hospitalized with suspected or confirmed SARS-CoV-2 infection. WHO Glob. Clin. Platf. COVID-19, no. July, p. abstract PEBLB20, 2021.

[12] Morina N, Kip A, Hoppen TH, Priebe S, Meyer T (2021). Potential impact of physical distancing on physical and mental health: a rapid narrative umbrella review of meta-analyses on the link between social connection and health. BMJ Open.

[13] The Ethiopian Public Health Institute, HIV Related Estimates, and Projections for Ethiopia–2017, no. March. Addis Ababa, Ethiopia, 2017.

[14] Worku ED, Asemahagn MA, Endalifer ML (2020). Epidemiology of HIV infection in the Amhara region of Ethiopia, 2015 to 2018 surveillance data analysis. HIV/AIDS - Res Palliat Care.

[15] Ethiopia Ministry of Health, National Comprehensive HIV Prevention, Care and Treatment Training for Health care Providers Participant Manual. Addis Ababa, Ethiopia, 2020.

[16] Tigabu BM, Agide FD, Mohraz M, Nikfar S (2020). Atazanavir / ritonavir versus lopinavir / ritonavir-based combined antiretroviral therapy (cART) for HIV-1 infection: a systematic review and meta-analysis. Afr Health Sci.

[17] Kanters S (2017). Comparative efficacy and safety of second-line antiretroviral therapy for treatment of HIV/AIDS: a systematic review and network meta-analysis. Lancet HIV.

[18] Kroenke K, Spitzer RL, Williams JBW (2001). Validity of a brief depression severity measure, the PHQ-9. JGIM.

[19] Gelaye B, Williams MA, Lemma S, Deyessa N, Bahretibeb Y, Shibre T, Wondimagegn D, Lemenhe A, Fann JR, Stoep AV, Zhou X-HA (2013). Validity of the patient health questionnaire-9 for depression screening and diagnosis in East Africa. Psychiatry Res.

[20] Nyblade L, Kerry M. Can we measure HIV stigma and discrimination? 2006.

[21] Zeng C (2018). A structural equation model of perceived and internalized stigma, depression, and suicidal status among people living with HIV / AIDS. BMC Public Health.

[22] Steffen O (2006). Negative life events, social support and gender difference in depression. Soc Psychiatry Psychiatr Epidemiol.

[23] Heslop K, Ross C, Osmond B, Wynaden D (2013). The Alcohol Smoking and Substance Involvement Screening Test (ASSIST) in an Acute Mental Health Setting. Int J Ment Health Addict.

[24] Bentler PM, Chou C-P (1987). Practical issues in structural modeling. Sociol Methods Res.

[25] Kinyanda E, Kuteesa M, Scholten F, Mugisha J, Baisley K, Seeley J, et al. Risk of major depressive disorder among older persons living in HIV-endemic central and southwestern Uganda. AIDS Care. 2016;119(601). 10.1080/09540121.2016.1191601 27263868

[26] Bitew T (2014). prevalence, and risk factors of depression in Ethiopia : a review. Ethiop J Health Sci.

[27] Kim MH (2015). Factors associated with depression among adolescents living with HIV in Malawi. BMC Psychiatry.

[28] Aguocha CC, Uwakwe RR, Duru CB, Diwe KK (2015). Prevalence and socio-demographic determinants of depression among patients attending HIV / AIDS Clinic in a Teaching Hospital in Imo State, Nigeria. Am J Med Sci Med.

[29] Holloway IW (2017). Network support, technology use, depression, and ART adherence among HIV-positive MSM of color.

[30] Tymchuk S, Gomez D, Koenig N, Gill MJ, Fujiwara E, Power C (2018). Associations between depressive symptomatology and neurocognitive impairment in HIV / AIDS. Can J Psychiatry.

[31] Tesfaw G, Ayano G, Awoke T, Assefa D, Birhanu Z, Miheretie G (2016). Prevalence and correlates of depression and anxiety among patients with HIV on- follow up at Alert Hospital, Addis Ababa. BMC Psychiatry.

[32] Mohammed M, Mengistie B, Dessie Y, Godana W. Prevalence of depression and associated factors among HIV patients seeking treatments in ART clinics at Harar Town, Eastern Ethiopia. J AIDS Clin Res. 2015;6(6).

[33] World Health Organization and C. University. Group interpersonal therapy (IPT) for depression (WHO generic field-trial 1.0). World Health Organ. 2016;1:100.

[34] Brandt R (2010). The mental health of people living with HIV / AIDS in Africa : a systematic. Afri J AIDS Res.

[35] Thomas PA, Liu H, Umberson D (2017). Family relationships and well-being. Innov Aging.

[36] Scheurer D, Choudhry N, Swanton KA, Matlin O, Shrank W. Association between different types of social support and medication adherence. Am J Manag Care. 2012;18(12). 23286676

[37] Li XM, Yuan XQ, Rasooly A, Bussell S, Wang JJ, Zhang WY (2018). An evaluation of impact of social support and care-giving on medication adherence of people living with HIV/AIDS a nonrandomized community intervention study. Medicine (United States).

[38] George S, McGrath N (2019). Social support, disclosure and stigma and the association with non-adherence in the six months after antiretroviral therapy initiation among a cohort of HIV-positive adults in rural KwaZulu-Natal, South Africa*. AIDS Care Psychol Socio-Medical Asp AIDS/HIV.

[39] Atukunda EC (2017). Understanding patterns of social support and their relationship to an ART adherence intervention among adults in rural southwestern Uganda. AIDS Behav.

[40] Liu C, Huang N, Fu M, Zhang H, Feng XL, Guo J (2021). Relationship between risk perception, social support, and mental health among general chinese population during the covid-19 pandemic. Risk Manag Healthc Policy.

[41] Li F (2021). Effects of sources of social support and resilience on the mental health of different age groups during the COVID-19 pandemic. BMC Psychiatry.

[42] Guo K (2021). Assessing social support impact on depression, anxiety, and stress among undergraduate students in Shaanxi province during the COVID-19 pandemic of China. PLoS ONE.

[43] Yimam W, Wedajo S, Prema K (2017). Magnitude and determinants of diabetes mellitus among people living with HIV in Dessie Referral hospital, North east Ethiopia. Int J Recent Adv Multidiscip Res.

[44] Shambel W, Kumara P, Yimam W, Molla A (2017). Hypertension as a silent killer: enormity definitive factors hypertension as a silent killer: enormity and definitive factors among habitant living with retrovirus. Int J Curr Med Sci.

[45] Id KS (2018). Low prevalence of depressive symptoms among stable patients on antiretroviral therapy in Johannesburg, South Africa. PLoS ONE.

[46] Bernard C, De Rekeneire N (2017). Prevalence and factors associated with depression in people living with HIV in sub- Saharan Africa : a systematic review and meta-analysis. PLoS ONE.

[47] Garey L, Bakhshaie J, Sharp C, Neighbors C, Zvolensky MJ, Gonzalez A (2015). Anxiety, depression, and HIV symptoms among persons living with HIV / AIDS : the role of hazardous drinking. AIDS Care.

[48] Mohamed II, Ahmad HEK, Hassaan SH, Hassan SM (2020). Assessment of anxiety and depression among substance use disorder patients: a case-control study. Middle East Curr Psychiatry.

[49] Ganguli M, Dodge HH, Mulsant BH (2002). Drug use and the risk of major depressive disorder, alcohol dependence, and substance use disorders. Arch Gen Psychiatry.

